# Complete Genome Sequence of vB_EcoM-UFV13, a New Bacteriophage Able To Disrupt *Trueperella pyogenes* Biofilm

**DOI:** 10.1128/genomeA.01292-16

**Published:** 2016-12-08

**Authors:** Vinícius S. Duarte, Roberto S. Dias, Andrew M. Kropinski, Pedro M. P. Vidigal, Flávia O. Sousa, André S. Xavier, Cynthia C. Silva, Sergio O. de Paula

**Affiliations:** a Department of Microbiology, Universidade Federal de Viçosa, Viçosa, Minas Gerais, Brazil; b Department of General Biology, Universidade Federal de Viçosa, Viçosa, Minas Gerais, Brazil; c Departments of Food Science, Molecular and Cellular Biology and Pathobiology, University of Guelph, Guelph, Ontario, Canada; d Núcleo de Análise de Biomoléculas (NuBioMol), Biological and Health Science Center, Universidade Federal de Viçosa, Viçosa, Minas Gerais, Brazil; e Department of Phytopathology, Universidade Federal de Viçosa, Viçosa, Minas Gerais, Brazil

## Abstract

vB_EcoM-UFV13, a member of the *T4virus* genus, shows lytic activity against *Escherichia coli* and effectiveness in controlling the biofilm formed by *Trueperella pyogenes*, which qualifies it as a promising component of phage cocktails for mastitis and metritis control.

## GENOME ANNOUNCEMENT

Metritis and mastitis are diseases in dairy cattle and can lead to economic losses of U.S.$2 billion annually in the United States (1–3). Common to both diseases, *Escherichia coli* and *Trueperella pyogenes* (formerly *Arcanobacterium pyogenes* [4]) have the ability to produce biofilm and with regard to metritis, there is a temporal relationship between both microorganisms found in the uterus. *E. coli* is the first pathogen that establishes in the uterus and is responsible for providing an appropriate intrauterine environment for development of *T. pyogenes* (1, 5). Thus, it is essential to find new agents that are effective in the control of both microorganisms.

vB_EcoM-UFV13 was isolated using a wild strain (*E. coli* 30) obtained from a dairy cow with clinical mastitis. The phage has a capsid with icosahedral symmetry (72 nm of length and width) and a 30 nm long contractile tail, thus indicating it as a member of the *Caudovirales* order, family *Myoviridae*. This virus showed lytic activity against *E. coli*, as well as being effective in the control of biofilm formed by *T. pyogenes*, which qualifies it as a promising component of phage cocktails for mastitis and metritis control. The bacteriophage was concentrated and purified using polyethylene glycol 8,000 and its genome extracted by proteinase K/phenol method (6).

The phage DNA was sent to Macrogen (South Korea) and the sequencing performed by Illumina Hiseq 2500 paired-end platform, with read quality evaluated by HCS (HiSeq Control Software v2.2.38) and RTA softwares (Real Time Analysis. v1.18.61.0). The sequence data was *de novo* assembled using SeqMan NGen 12 (DNAStar, Madison, WI, USA) software. The consensus sequence was opened to be collinear with *Yersinia* phage PST and the fragments were reassembled using SeqMan Pro 12, into a single contig of 165,771 bp (570-fold coverage) with a G+C content of 34.8%. 269 open reading frames (ORFs) were predicted and annotated using the MyRast program (7) with 193 ORFs encoding hypothetical proteins, 39 ORFs encoding structural proteins, and the majority (228 ORFs) transcribed from the negative strand. Notable is the presence of ORF 130, which encodes a glycoside hydrolase (family 24), an enzyme able to act in a Gram-positive biofilm and cause lysis in a Gram-negative bacteria. This property was also observed by the authors of reference (8). A gene cluster, without introns or pseudogenes, encoding 10 tRNAs (Gln, Leu, Gly, Pro, Ser, Thr, Met, Tyr, Asn, and Arg) was identified using tRNAscan-SE (5, 9). The genome of UFV13 is related to *Escherichia, Shigella*, and *Yersinia* phages belonging to the *T4virus* genus. Their collinearity was confirmed by progressiveMauve analysis (10).

The next steps of this work will be focused on the interaction between phage UFV13 and *Trueperella pyogenes*, using proteomic and transcriptomic approaches.

### Accession number(s).

The whole-genome sequence generated has been deposited at GenBank under the accession no. KU867876.
